# Understanding Urban Mobility Among Wheelchair Users: The Intersection of Physical Impairment, Wheelchair Mobility Proficiency, and Self-Efficacy in Wheeled Mobility—A Scoping Review

**DOI:** 10.3390/healthcare14162517

**Published:** 2026-08-12

**Authors:** Seyedeh Hiva Houshyar Yazdian, Karina Santos Guedes de Sá, Wolfgang Jacquet, Johan Stiens, Yves Vanlandewijck

**Affiliations:** 1 Department of Educational Sciences, Faculty of Psychology and Educational Sciences, Vrije Universiteit Brussel, Pleinlaan 2, 1050 Brussels, Belgium; wolfgang.jacquet@vub.be; 2 Department of Electronics and Informatics, Vrije Universiteit Brussel, Pleinlaan 2, 1050 Brussels, Belgium; johan.stiens@vub.be; 3 Laboratory of Physical Assessment, Exercise and Adapted Sport, School of Physical Education, Graduate Program in Physical Education, State University of Campinas, Campinas 13083-851, Brazil; karina-sa@outlook.com; 4 Department of Clinical Sciences KLIM, Faculty of Medicine and Pharmacy, Vrije Universiteit Brussel, Pleinlaan 2, 1050 Brussels, Belgium; 5 Faculty of Movement and Rehabilitation Science, KU Leuven, Tervuursevest 101, 3001 Leuven, Belgium; yves.vanlandewijck@kuleuven.be; 6 Department of Physiology, Nutrition and Biomechanics, Swedish School of Sport and Health Sciences (GIH), SE-114 86 Stockholm, Sweden

**Keywords:** manual wheelchair users, urban accessibility, built environment, wheelchair mobility skills, international classification of functioning, disability and health

## Abstract

**Highlights:**

- No included study jointly assessed physical impairment, self-efficacy in wheeled mobility, and wheelchair mobility proficiency.
- Greater physical impairment was generally associated with more barriers and effort; self-efficacy affected task engagement, while mobility proficiency affected barrier negotiation.
- Urban accessibility should be understood as a person–wheelchair–environment interaction, rather than solely through infrastructure compliance.
- Research and practice should integrate multidimensional assessment, infrastructure adaptation, wheelchair-skills training, self-efficacy support, and access to well-fitted wheelchairs.

**Abstract:**

**Background/Objectives**: Urban accessibility is a key determinant of participation for manual wheelchair users, yet accessibility experiences vary according to physical impairment, self-efficacy in wheeled mobility, and wheelchair mobility proficiency. This scoping review examined urban accessibility challenges in relation to these three dimensions. **Methods**: Following PRISMA-ScR guidelines, eight databases were searched for English-language peer-reviewed studies published from January 2015 to May 2025. Eligible studies addressed outdoor or urban mobility challenges among manual wheelchair users in relation to at least one dimension. Two reviewers independently screened records, assessed full texts, and extracted data using a standardized form. Findings were synthesized within an International Classification of Functioning, Disability and Health-informed framework. Fourteen studies were included. **Results**: The evidence base was methodologically diverse and fragmented; no study examined all three dimensions simultaneously, and wheelchair mobility proficiency received the least attention. Greater physical impairment was associated with more perceived barriers and higher effort. Self-efficacy influenced avoidance of or engagement in mobility tasks beyond physical functioning. Wheelchair mobility proficiency, supported by training, experience, and wheelchair fit, was associated with managing ramps, uneven surfaces, and other environmental demands, but was rarely assessed using standardized performance measures. **Conclusions**: Urban accessibility reflects interactions among physical functioning, self-efficacy, wheelchair mobility proficiency, assistive-device characteristics, and environmental conditions. Accordingly, urban mobility should be understood as a person–wheelchair–environment interaction rather than solely as an interaction between the individual and the built environment. Integrative studies using standardized measures and more systematic reporting of wheelchair fit and configuration are needed to inform impairment-inclusive urban planning, rehabilitation, and policy.

## 1. Introduction

Urban mobility for users of manual wheelchairs emerges from the dynamic interaction between body functions and characteristics, activity performance, participation needs, and contextual dimensions, as structured by the International Classification of Functioning, Disability and Health (ICF) [1]. For manual wheelchair users, this interaction is mediated by the wheelchair itself; therefore, urban mobility can be understood as a person–wheelchair–environment system. From the ICF perspective, safe and independent participation in the urban environment depends simultaneously on body functions and structures, activities and participation, and environmental and personal dimensions that shape behavior and actual performance. In the case of users of manual wheelchairs, body functions and structures are related to the type and level of physical impairment (PI); the activities and participation component refers to wheelchair mobility proficiency and the execution of daily tasks; personal dimension encompasses aspects such as self-efficacy and confidence in mobility, whereas environmental dimension includes characteristics of urban infrastructure, such as sidewalk quality, slopes, curbs, obstacles, and accessibility of public spaces, as well as wheelchair-related factors such as fit and configuration [2,3,4,5,6].

In this context, for manual wheelchair users in particular, everyday activities such as commuting, accessing public services, or engaging in social and recreational life depend heavily on the accessibility of sidewalks, crossings, public transportation, and buildings [7,8,9,10,11,12,13]. Despite international accessibility standards and growing policy attention, many urban spaces continue to present physical, organizational, and contextual barriers that limit safe and independent mobility for wheelchair users [7,12,14].

Understanding the interactions between PI, wheelchair mobility proficiency, self-efficacy, and urban infrastructure is fundamental to explaining the full participation of manual wheelchair users in daily life. Although urban infrastructure constitutes an extrinsic environmental factor to the individual and, therefore, cannot be directly modified by the individual, intrinsic factors, such as the type and level of PI, wheelchair mobility proficiency, and self-efficacy in facing mobility challenges, influence how this environment is perceived and experienced. Consequently, compliance with formal accessibility standards does not necessarily ensure that an urban feature is safe, usable, or manageable for every wheelchair user. Thus, the same urban context can represent different levels of barrier or facilitator to mobility, depending on individual characteristics [7,15].

PI is typically operationalized through functional limitations, and evidence suggests that perceived barriers differ across wheelchair users depending on function-related factors [16,17,18]. Self-efficacy in wheeled mobility refers to a person’s perceived ability or confidence to perform wheelchair-related mobility tasks and manage environmental challenges. It influences whether individuals attempt or avoid specific mobility tasks, even when they have the functional capacity to perform them [5,19]. Wheelchair mobility proficiency, often reflected in maneuvering ability and shaped by training and experience, can influence how users negotiate environmental barriers (e.g., slopes or ramps, curbs, uneven terrain) and perform tasks such as public-transit entering or exiting and on-board maneuvering [20,21,22].

Although the interaction between individual capacity and environmental demands has long been recognized in person–environment fit models [23], this perspective remains insufficiently integrated in recent literature on manual wheelchair users’ urban mobility. In particular, it remains unclear how PI, self-efficacy in wheeled mobility, and wheelchair mobility proficiency have been jointly conceptualized and measured. Each of these dimensions has been examined in prior research, but the existing literature remains fragmented. Studies vary widely in how PI is defined and reported, ranging from detailed clinical descriptors to broad self-reported categories [18,24], while others omit physical profiling information altogether. Self-efficacy and wheelchair mobility proficiency are frequently embedded within broader frameworks such as physical activity participation, accessibility modeling, or transportation usability [2,3,6,25,26,27,28,29]. However, reporting and measurement vary across studies. Moreover, few studies consider these dimensions jointly, limiting the ability to understand how PI, self-efficacy, and wheelchair mobility proficiency converge, shaping real-world urban mobility experiences for manual wheelchair users.

Given the heterogeneity of study designs, populations, environments, and outcome measures, a scoping review is an appropriate approach to systematically map the existing evidence, identify key concepts, and highlight knowledge gaps. Scoping reviews are particularly suited to areas where research is emerging, methodologically diverse, and conceptually complex [30,31,32]. The aim of this scoping review was to examine how urban accessibility challenges differ for manual wheelchair users based on PI, self-efficacy in wheeled mobility, and wheelchair mobility proficiency. Specifically, the review sought to (1) map how these three dimensions are conceptualized and measured in literature, (2) summarize reported accessibility challenges and facilitators associated with each dimension, and (3) identify gaps to guide future research and urban accessibility interventions.

## 2. Materials and Methods

### 2.1. Study Design

This study is a scoping review designed to identify, analyze, and synthesize the available literature on urban accessibility challenges faced by manual wheelchair users, with particular attention to how these challenges vary based on PI, self-efficacy in wheeled mobility, and wheelchair mobility proficiency. The review followed the Preferred Reporting Items for Systematic Reviews and Meta-Analyses extension for Scoping Reviews (PRISMA-ScR) guidelines [32]. The protocol was registered on the Open Science Framework (OSF) prior to study commencement (https://osf.io/hp2xz (access on 22 July 2026)).

### 2.2. Eligibility Criteria (PCC Framework)

The review was guided by the PCC framework (Population, Concept, Context): The study population (P) consisted of manual wheelchair users, including terms such as “manual wheelchair users”, “wheelchair users”, and “people with physical disabilities”. The study concept (C) encompassed urban accessibility challenges, user perception, impairment severity, and wheelchair mobility proficiency, represented by keywords such as “accessibility challenges”, “barriers”, “user confidence”, “wheelchair skills”, and “self-efficacy”. The context (C) referred to the urban environment, including the built environment and public spaces, with examples such as “urban environment”, “built environment”, “public spaces”, and “urban infrastructure”.

### 2.3. Inclusion and Exclusion Criteria

All articles that met the following criteria were included in this review: I—Studies focusing on manual wheelchair users; II—Studies that investigate mobility challenges in urban settings; III—Studies examining the role of PI, self-efficacy in wheeled mobility, or wheelchair mobility proficiency; IV—All study designs (qualitative, quantitative, or mixed-methods); V—Articles published in English; VI—Peer-reviewed journal articles; VII—Articles published between January 2015 and May 2025.

All articles that met one of the following criteria were excluded in this review: I—Studies involving only powered or electric wheelchair users; II—Studies limited to indoor environments (e.g., homes, schools, hospitals); III—Studies focused on the access to the facilities (e.g., entrance of the buildings) and no focus on mobility; IV—Studies not involving participants with physical disabilities; V—Editorials, opinion pieces, conference abstracts without full text; VI—Review articles (e.g., systematic reviews or scoping reviews).

These exclusions were applied to maintain consistency with the predefined review question, which focused specifically on manual wheelchair users navigating outdoor urban environments. Studies involving only powered-wheelchair users, indoor mobility, or access to individual facilities without a broader mobility component were therefore considered outside the scope of the review.

### 2.4. Information Sources and Search Strategy

Searches were conducted in the following electronic databases: PubMed (MEDLINE), Scopus, Web of Science, Embase, PsycINFO, and the Publicly Available Content Database via ProQuest, SportDiscus, and Education Full Text (H.W. Wilson).

A search strategy combining controlled vocabulary (e.g., MeSH terms) and free-text terms was applied. Boolean operators (AND, OR) were used to connect search terms related to wheelchair users, urban accessibility, and user-related dimensions such as skill, PI, and confidence. As a representative example, the complete PubMed search strategy was:

(wheelchair* OR “wheelchair user*” OR “people with physical disabilit*” OR “manual wheelchair” OR “mobility aid user*” OR “wheelchair-bound” OR “persons with physical disabilities” OR “Health Services for Persons with Disabilities” [MeSH] OR “Persons with Disabilities” [MeSH] OR “Wheelchairs” [MeSH]) AND (“accessibility challenges” OR “barriers” OR “user confidence” OR “wheelchair skills” OR “self-efficacy” OR “accessibility barrier*” OR “urban barrier*” OR “navigation difficulty” OR “mobility challenge*” OR “environmental barrier*” OR “obstacle*” OR “confidence” OR “skill proficiency” OR “time since injury” OR “functional limitation*” OR “impairment level” OR “disability severity” OR “facilitators” OR “environmental effects” OR “route selection” OR “surface” OR “terrain” OR “slope” OR “confidence-based” OR “confidence model” OR “accessibility assessment” OR “accessibility assessment” OR “pedestrian network” OR “accessibility mapping” OR “outdoor accessibility” OR “safe navigation” OR “accessible path*” OR “social participation” OR “Self Efficacy” [MeSH] OR “Motor Skills” [MeSH] OR “Activities of Daily Living” [MeSH] OR “Motor Skills Disorders” [MeSH]) AND (“urban environment*” OR “built environment” OR “public space*” OR “urban infrastructure” OR “urban area*” OR “city” OR “cities” OR “urban mobility” OR “streetscape” OR “trail” OR “forest” OR “park” OR “outdoor” OR “Urban Health” [MeSH] OR “Cities” [MeSH] OR “Public Facilities” [MeSH] OR “Environment Design” [MeSH]).

The full search log, including database-specific search strings, search dates, and the number of records retrieved per database, is provided in Supplementary Table S1 (Search Log Table). Search results were exported to a reference management tool (Zotero), and duplicates were removed prior to screening.

### 2.5. Study Selection

Two independent reviewers (HH and KS) screened the titles and abstracts of all identified records against the predefined eligibility criteria. Full texts of potentially relevant studies were subsequently retrieved and independently assessed for eligibility by both reviewers. Discrepancies at either stage of the selection process between reviewers were resolved through discussion or consultation with a third reviewer (YV).

**Figure 1:**
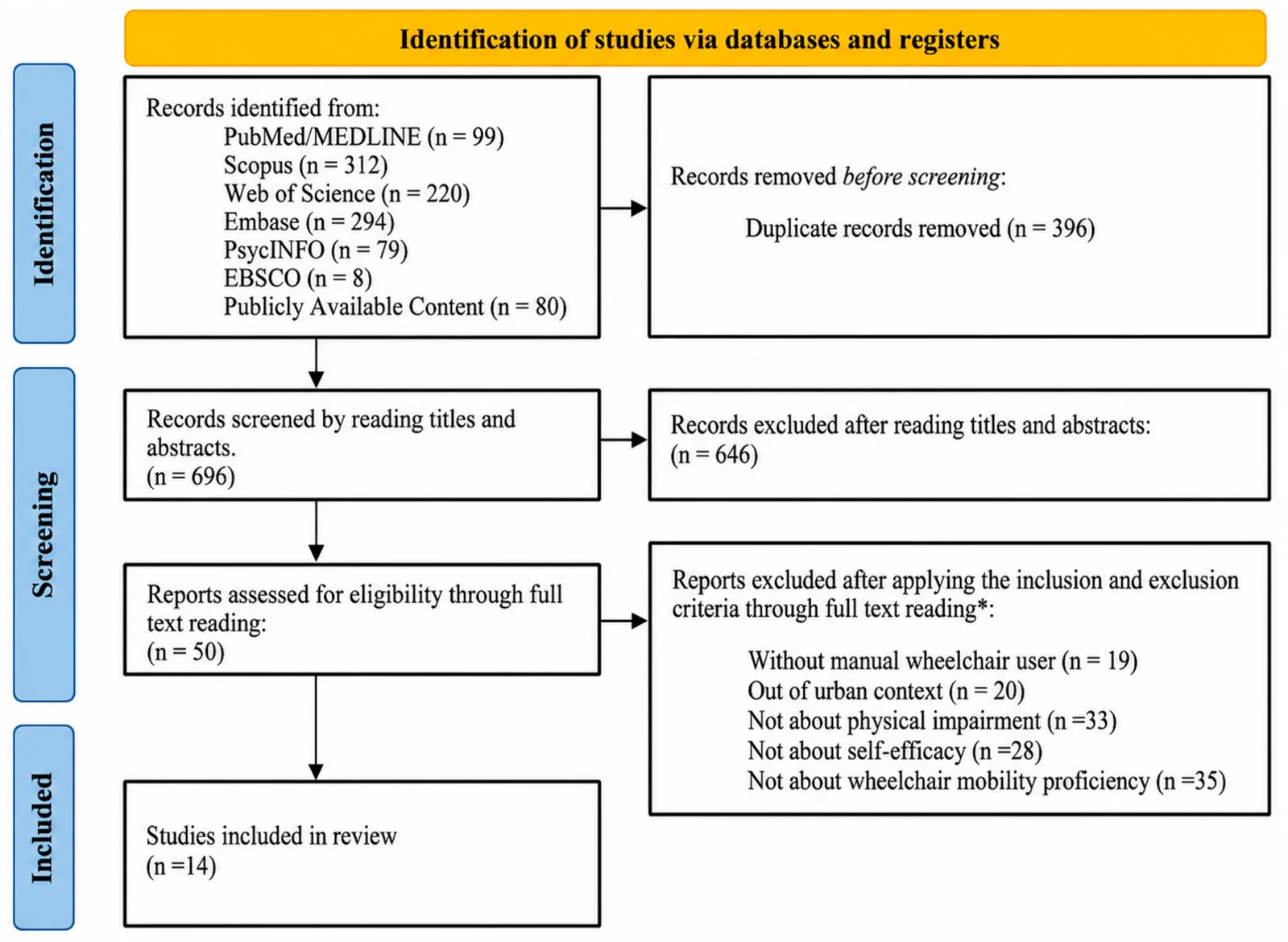
PRISMA Flow Diagram. * Articles were excluded if they met at least one of the exclusion criteria. We recorded all criteria applicable to each article; some met more than one criterion. Therefore, the total number of exclusions is greater than the number of articles analyzed. Source: Page MJ, et al. BMJ 2021; 372: n71. doi: 10.1136/bmj.n71 [33].

The search and screening process resulted in 14 studies being included in this scoping review. The relatively small number of included studies reflects the specificity of the review question: eligible studies had to address manual wheelchair users, outdoor or urban mobility, and at least one of the three user-level dimensions. The search strategy was intentionally broad to maximize sensitivity, resulting in the retrieval of many records that did not meet all eligibility criteria. The detailed selection process, including reasons for exclusion at the full-text stage, is shown in the PRISMA Flow Diagram (Figure 1).

### 2.6. Data Extraction

Data from included studies were extracted using a standardized data extraction form. Extracted information included: Author and year of publication, study objective, sample (age, gender, type of impairment, wheelchair type), urban context and settings, accessibility challenges identified, findings related to PI, self-efficacy, and wheelchair mobility proficiency. The extraction form also included fields for ergonomic or device-related factors and associated findings, such as wheelchair type, fit, seating adequacy, stability, and configuration, when these were reported by the included studies. However, these factors were neither explicit eligibility criteria nor among the three primary review dimensions and were therefore not emphasized to the same extent in the synthesis. The complete extracted dataset for all included studies is provided in Supplementary Table S2 (Study extraction table).

### 2.7. Data Charting and Synthesis

Data were synthesized using a convergent integrated approach [34] within a deductive framework informed by the ICF. Extracted data were first charted by study characteristics, population, urban context, environmental barriers and facilitators, and reported user-level dimensions. In this review, user-level dimensions included PI, self-efficacy in wheeled mobility, and wheelchair skill proficiency.

Qualitative findings and qualitative components of mixed-methods studies were quantified at the study level by transforming narrative findings into categorical indicators. These indicators included whether each study addressed each review dimension and which types of barriers or facilitators were reported. These categorical codes were then merged with quantitative findings, including quantitative components of mixed-methods studies, to support descriptive numerical mapping and narrative synthesis.

Findings were subsequently interpreted in relation to relevant ICF domains, including body functions and structures, activities and participation, personal factors, and environmental factors. We also examined whether studies considered the three user-level dimensions independently or in combination with environmental barriers and facilitators.

To clarify how the ICF informed data charting and synthesis, Table 1 presents the coding framework used to map the review dimensions to relevant ICF components. This framework was used to distinguish between body-function-related characteristics, activity and participation dimensions, personal factors, and environmental or assistive-device factors during data extraction and synthesis.

### 2.8. Methodological Quality Assessment of the Studies

The methodological quality of the included studies was assessed using the JBI Critical Appraisal Tools, selected according to the design of each study. The application of the tools followed the official JBI guidelines, considering items such as clarity of objectives, adequacy of methodological design, rigor in data collection and analysis, identification and control of potential biases, and transparency in the presentation of results. Each study was independently evaluated by two reviewers, and any disagreements were resolved by consensus. No overall numerical appraisal scores were calculated; instead, item-level judgments were recorded as Yes, No, Unclear, or Not applicable. The appraisal findings were not used to exclude studies or formally weight their results. Instead, they were used to contextualize the strength of the evidence and to identify findings requiring more cautious interpretation, particularly where studies showed unclear participant selection, limited consideration of confounding, or insufficient reporting of researcher reflexivity.

## 3. Results

### 3.1. Quality Assessment of the Studies

Methodological quality was assessed to describe the reporting quality and robustness of the included evidence, not to exclude studies. Cross-sectional studies generally used appropriate measures and statistical analyses, but several had unclear inclusion criteria or limited consideration of confounding. Qualitative studies were generally congruent with their research questions and methods, although researcher reflexivity was often insufficiently reported. Accordingly, findings from these studies were retained in the evidence map but interpreted cautiously, particularly when drawing broader conclusions across heterogeneous populations and study designs. Full appraisal results are provided in Supplementary Table S3.

### 3.2. Study Characteristics

At the end of the screening and selection process, 14 studies were included, each addressing at least one of the three central aspects of this review: PI, self-efficacy in wheeled mobility, and wheelchair mobility proficiency. None of the studies examined all three aspects simultaneously, highlighting a significant gap in international literature, i.e., a holistic approach of the manual wheelchair user moving around in an urban environment. The evidence was also unevenly distributed across the three dimensions: eight studies addressed PI, six addressed self-efficacy in wheeled mobility, and only three provided findings directly related to wheelchair mobility proficiency. Consequently, the evidence regarding wheelchair mobility proficiency should be interpreted more cautiously, as it was based on a substantially smaller number of studies.

Table 2 summarizes the methodological designs and the criteria addressed by each study. The 14 studies included were methodologically diverse, including observational studies, qualitative investigations, feasibility pilots, mixed-methods designs, and simulation-based or Geographic Information Systems (GIS)-based modeling approaches. The included evidence comprised both participant-based empirical studies and modeling or methodological studies. The latter did not directly investigate wheelchair users’ lived mobility experiences but demonstrated potential applications for accessibility assessment and route planning. Most studies addressed only one of the three criteria, indicating that the three review dimensions were usually examined separately rather than in combination.

Geographically, the studies were conducted across 10 countries, most frequently in Canada (5 studies [27,28,29,39,42]), followed by the United States (2 studies [25,26]). The remaining countries each contributed one study: Kenya and the Philippines (one multi-country study [35]), Spain [36], Mexico [37], Mongolia [38], Denmark [16], India [40], and Saudi Arabia [41].

**Figure 2:**
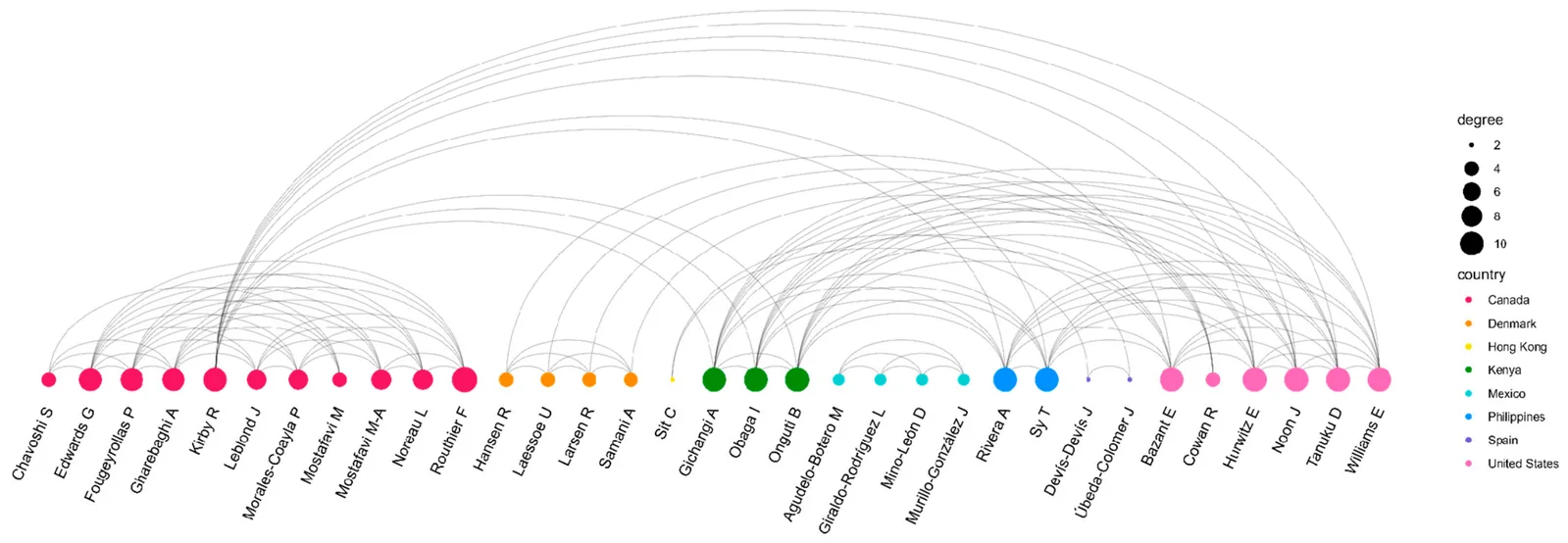
Distribution of scientific collaborations between authors and countries. Network of connections between authors, where each node represents a person, colored according to their country of affiliation and size (degree) according to their degree of connectivity. The lines indicate collaborations between the participants.

To complement this analysis, a co-authorship network was constructed based on the 14 included studies (Figure 2) to visualize collaborative relationships among authors and identify their national affiliations. The studies included in this review are characterized by small, predominantly national collaborative groups, with only a few authors serving as links across countries. Only 2 of the included studies had authors affiliated with more than 2 countries [35,38].

Sample sizes ranged from 3–2041 participants (median: 127); one methodological field-audit study [27] did not include an empirical participant sample. Participant populations included adults (≥18 years), with reported ages spanning 18 to 90 years across studies that provided age information [16,27,36,37,38,40,41]. Age was not reported in five studies [25,26,29,42]. Where reported, samples were generally mixed-gender; gender distribution was not reported in five studies [25,28,29,40,42].

Recruitment settings were primarily community- and organization-based, often using convenience and purposive sampling through impairment and rehabilitation centers and Non-Governmental Organizations (NGOs) [28,35,38,39,40], or online survey dissemination via disability organizations and support centers [16,26,41]. Additional settings included university impairment services [36] and a nationally representative household survey [37], while one study used naturalistic observation in public transit without active recruitment [25]. Three modeling and tool studies did not involve participant recruitment [27,29,42].

Impairment characteristics were variably reported, ranging from broad impairment categories to detailed neurological injury profiles; two studies did not report impairment specifics [25,28]. Several studies provided diagnosis and type distributions, including mixed etiologies among wheelchair users [35], broad impairment-category classifications with grading systems [36], and national survey data emphasizing mobility impairment and self-reported severity [37]. Other studies reported primary conditions such as Spinal Cord Injury (SCI) and cerebral palsy alongside other neurological and musculoskeletal conditions [26], or predominant physical disabilities with smaller sensory and mental health groups [41]. Among studies focusing on SCI populations, clinical reporting varied and was often limited to injury level, completeness, or duration, with inconsistent documentation of etiology and detailed descriptors [16,38]. In several cases, only duration of impairment, wheelchair-use history, or broad mobility classifications were provided rather than comprehensive clinical detail [27,39,40,41,42].

Climate and weather considerations were reported in 7 of the 14 included studies [26,27,28,38,39,40,42]. In Canada, multiple studies incorporated snow into mobility assessment or routing models [27,28] and one intervention study included weather as an explicit obstacle within a barrier self-efficacy measure [39]. Weather was also reported as a practical barrier in Mongolia [38], wet and icy surfaces were explicitly linked to mobility difficulty in the United States [26], and monsoon-related water-logging and intense sun were highlighted in India [40]. The remaining studies did not report climate or weather considerations [16,25,29,35,36,37,41].

### 3.3. Physical Impairment

Among the 14 studies included in this review, eight (57.14%) explicitly addressed the level of PI or provided stratified information that allowed severity-related interpretations [16,25,35,36,37,38,40,41]. Across several studies, higher levels of PI were associated with greater perceived barriers across multiple domains, including environmental accessibility, transportation, physical activity participation, and daily mobility.

Considering the definitions of the studies included in this review, for the level of PI, we identified that in Mexico, individuals with “moderate impairment” were more than twice as likely to report barriers compared to those with “mild impairment” (OR 2.39; 95% CI 1.6–3.5), while those with “severe impairment” were more than four times as likely to report barriers (OR 4.58; 95% CI 3.2–6.6) [37]. Similarly, in Spain, students with “higher levels of PI” reported a greater number and intensity of barriers, especially intrapersonal ones [36]. The level of PI was also related to ergonomic and mobility challenges, such as difficulties using buses, inadequate devices, and limitations in daily activities, especially among users of manual wheelchairs and people with “more complex disabilities”, as observed in different contexts [25,35,38,40,41]. However, this pattern was not consistent across all studies. Among users of manual wheelchairs in Denmark, the level or completeness of spinal cord injury was not associated with any subdomain of barriers after environmental and ergonomic adjustments [16].

### 3.4. Self-Efficacy in Wheeled Mobility

Six studies (42.86%) addressed self-efficacy in wheeled mobility [27,28,29,36,39,42]. Across studies, self-efficacy was primarily conceptualized as perceived ability to perform mobility tasks. Several studies associated self-efficacy with task engagement, avoidance, or modeled accessibility, indicating that perceived ability may shape mobility behavior even when a person has the functional capacity to perform a task [27,28,29,42]. In accessibility mapping and routing applications, self-efficacy was translated into a difficulty score for each sidewalk segment, indicating how hard each segment would be for a given user [42]. In contrast, two studies focused on physical activity and framed self-efficacy at the individual level, either as a personal barrier to being active [36] or as self-efficacy to participate in leisure-time physical activity despite challenges such as transportation, weather, or pain [39].

In built-environment applications, self-efficacy was typically measured with the Wheelchair Use Confidence Scale (WheelCon), which asks users to rate their confidence on a 0–100 scale [27,28,29,42], and was operationalized as a segment-level difficulty or accessibility score (sometimes converted into fuzzy categories) to support accessibility modeling and user-specific routing. Lower confidence was associated with uneven surfaces, steep inclines, and obstacles, even when users’ level of functioning would otherwise allow them to perform the task. Wider, ramped, and unobstructed paths increased self-efficacy; higher confidence corresponded to higher modeled accessibility, speed, and reachable area, and to different “accessible routes” across individuals [27,28,29,42]. In physical-activity studies, self-efficacy was reported descriptively (mean 1.28/4 barrier rating) [36] and showed mixed change over time in an intervention context (no change T1–T2; decrease T2–T3) [39].

### 3.5. Wheelchair Mobility Proficiency

Only three (21.43%) of the included studies provided results directly related to wheelchair mobility proficiency, and together they highlighted how maneuvering skills, prior training, and device–environment interactions shape users’ ability to navigate urban and transport settings. Across these studies, wheelchair mobility proficiency emerged not as an isolated construct, but as a factor intertwined with environmental barriers, ergonomic constraints, and the adequacy of the mobility device. Table 3 describes the main characteristics of these studies.

In the study by Frost et al. (2015) [25], proficiency in skills was inferred from the ability to maneuver wheeled mobility devices during boarding and leaving buses. The study found that steeper ramp slopes, particularly those exceeding 9.5°, were associated with more incidents and assistance, while misaligned approach or departure angles greater than 30° were involved in several incidents. These findings suggest that both ramp slope and approach alignment may increase maneuvering demands, although wheelchair mobility proficiency was not formally measured. Williams et al. (2017) [35] noted that many users in Kenya and the Philippines had little or no formal training in wheelchair skills, learning by trial and error, which was associated with unsafe maneuvers and reduced independence, whereas those with more training reported greater self-efficacy. In a subsequent study by Frost et al. (2020) [26], proficiency was again indirectly assessed through self-reported difficulties, revealing that only one-third had received training in using public transport and that the lack of instruction was associated with greater challenges on steep ramps and on wet and uneven surfaces.

## 4. Discussion

From the perspective of the ICF, the findings of this review suggest that the urban mobility experience of manual wheelchair users is influenced by the relationship between PI, self-efficacy in wheeled mobility, wheelchair mobility proficiency, and environmental conditions. According to the included studies, higher levels of impairment were generally associated with greater perceived environmental and mobility-related barriers; higher levels of self-efficacy were associated with greater willingness to face urban mobility challenges; and wheelchair mobility proficiency emerged as an important factor in negotiating ramps, uneven surfaces, obstacles, and public transport.

These observations provide incremental refinement to, rather than substitution of, established person–environment models. The idea that environmental demands are experienced differently depending on individual capacity is well established in person–environment fit models. Lawton and Nahemow’s ecological model of aging conceptualizes functioning as the result of the fit between personal competence and environmental press. Applied to manual wheelchair mobility, this suggests that the same urban feature, such as a ramp, curb, slope, or uneven surface, may represent different levels of challenge depending on the user’s PI, wheelchair mobility proficiency, and self-efficacy [23].

Therefore, the novelty of this review is not the general concept of user–environment interaction, but the mapping of how these three user-level dimensions have been conceptualized, measured, and integrated in studies of manual wheelchair users’ urban mobility. One of the main findings of this review is that none of the included studies jointly examined PI, self-efficacy in wheeled mobility, and wheelchair mobility proficiency. This lack of integrated analysis limits a comprehensive understanding of how manual wheelchair users interact with and are influenced by urban environments. Without considering these dimensions together, current evidence captures only fragments of the user–environment relationship, missing the interdependence among functional capacity, personal factors, and real-world mobility performance.

These limitations become even more evident when considering broader structural determinants of accessibility. Complementary contextual literature outside the 14 included studies, including international policy reports, suggests that economic conditions shape the availability and quality of accessible infrastructure, which, in turn, influences participation opportunities for people with impairments [43,44,45,46]. In resource-constrained environments, poorer maintenance and limited access to public transport can exacerbate these challenges, particularly for users with more severe PI, lower self-efficacy, or reduced wheelchair mobility proficiency [43,47]. The limited integration of user-level dimensions with macro-level determinants highlights the need for interdisciplinary, internationally collaborative research that better captures accessibility as a multi-layered phenomenon.

The co-authorship network also shows that the evidence base is concentrated in small, predominantly national research groups, with limited cross-country collaboration. This may restrict the range of urban, policy, and rehabilitation contexts represented in the literature, reinforcing the need for broader international collaboration in future research.

Studies addressing PI generally suggested that greater functional impairment may be associated with a greater perception of environmental and mobility-related barriers. However, these associations should not be interpreted as deterministic, because users with similar levels of impairment may experience urban mobility differently depending on factors such as wheelchair fit, prior experience, mobility proficiency, self-efficacy, and environmental conditions. Greater functional limitations may also involve greater physical effort [48], more time [49], and greater energy expenditure to perform daily tasks and commutes. Users with more severe PI also appear more sensitive to wheelchair-related ergonomic limitations, especially when the wheelchair does not offer adequate trunk stability or does not facilitate functional reach. Since well-fitted wheelchairs can substantially improve efficiency and safety in daily activities, their absence tends to amplify perceived limitations [50,51]. Consequently, individuals with greater severity depend more heavily on environmental facilitators, such as adequate ramps, regular surfaces, and level transitions, to achieve basic autonomy and safety in the urban environment.

Self-efficacy, understood as an individual’s perceived ability to perform tasks and cope with challenges [52], may be an important personal factor for participation in daily activities, especially in contexts marked by environmental barriers. Within the included studies, higher levels of self-efficacy are associated with a greater willingness to face urban challenges [27,28,42]. The study by Fliess-Douer et al. (2013) [53] demonstrates a fair-to-moderate relationship between wheelchair mobility proficiency and perceived self-efficacy. This suggests that feeling capable and being capable are related, but not identical. Supporting literature further suggests that previous mobility experiences may strengthen or weaken self-efficacy over time [54]. Williams et al. (2017) and Frost et al. (2020) [26,35] also demonstrate that the absence of formal training reduces self-efficacy, while prior training experiences are associated with greater confidence, better decision-making, and fewer reported difficulties.

Within the limited included evidence, wheelchair mobility proficiency emerges as a central determinant for urban navigation among wheelchair users, directly influencing their ability to negotiate ramps, navigate obstacles, handle uneven surfaces, and use public transportation. Supporting literature suggests that this proficiency may reflect a combination of propulsion technique, postural control, prior experience, and ergonomic suitability of equipment, factors that affect both efficiency and safety during movement [49]. Despite its functional relevance and possible implications for social participation, wheelchair mobility proficiency is still poorly measured objectively, with few studies employing standardized instruments, such as the Wheelchair Skills Test, or performance-based assessments [55]. Greater use of feasible, standardized performance-based assessments could strengthen comparability across studies and clarify which specific skills matter most for safe and independent urban mobility [53].

In addition to the three core dimensions analyzed in this review, ergonomic factors related to wheelchair configuration and device–environment interaction emerged as a secondary contextual theme. However, these factors were not predefined as a separate review dimension and were not consistently assessed across the included studies. Complementary biomechanical literature outside the 14 included studies suggests that wheelchair fit and setup may influence physical effort, stability, and comfort, which is especially relevant for people with higher levels of PI [56,57]. Ergonomic adequacy may also interact with wheelchair mobility proficiency and self-efficacy in wheeled mobility, as an ergonomically adjusted wheelchair tends to enhance the feeling of control and safety during mobility, contributing to higher levels of self-efficacy [58,59]. Furthermore, ergonomic improvements may support technical performance in wheelchair maneuverability and obstacle negotiation [60,61,62,63,64,65,66,67]. However, ergonomic variables were rarely examined explicitly in the included studies, and when reported, they were often described as barriers (e.g., poor fit, insufficient seating, unstable devices), particularly for users with more complex PI or upper limb involvement [35,38,40]. Therefore, the available evidence does not permit firm conclusions about their independent contribution to urban mobility. This reinforces the need to consider assistive-device characteristics alongside user and environmental factors in future research.

Wheelchair maintenance and mechanical condition were not systematically reported in the included studies and, therefore, could not be synthesized in this review. Factors such as tire condition, wheel and caster function, brakes, bearings, and loose or damaged components should be considered in future urban mobility research because they may influence the practical performance of the person–wheelchair system.

**Figure 3:**
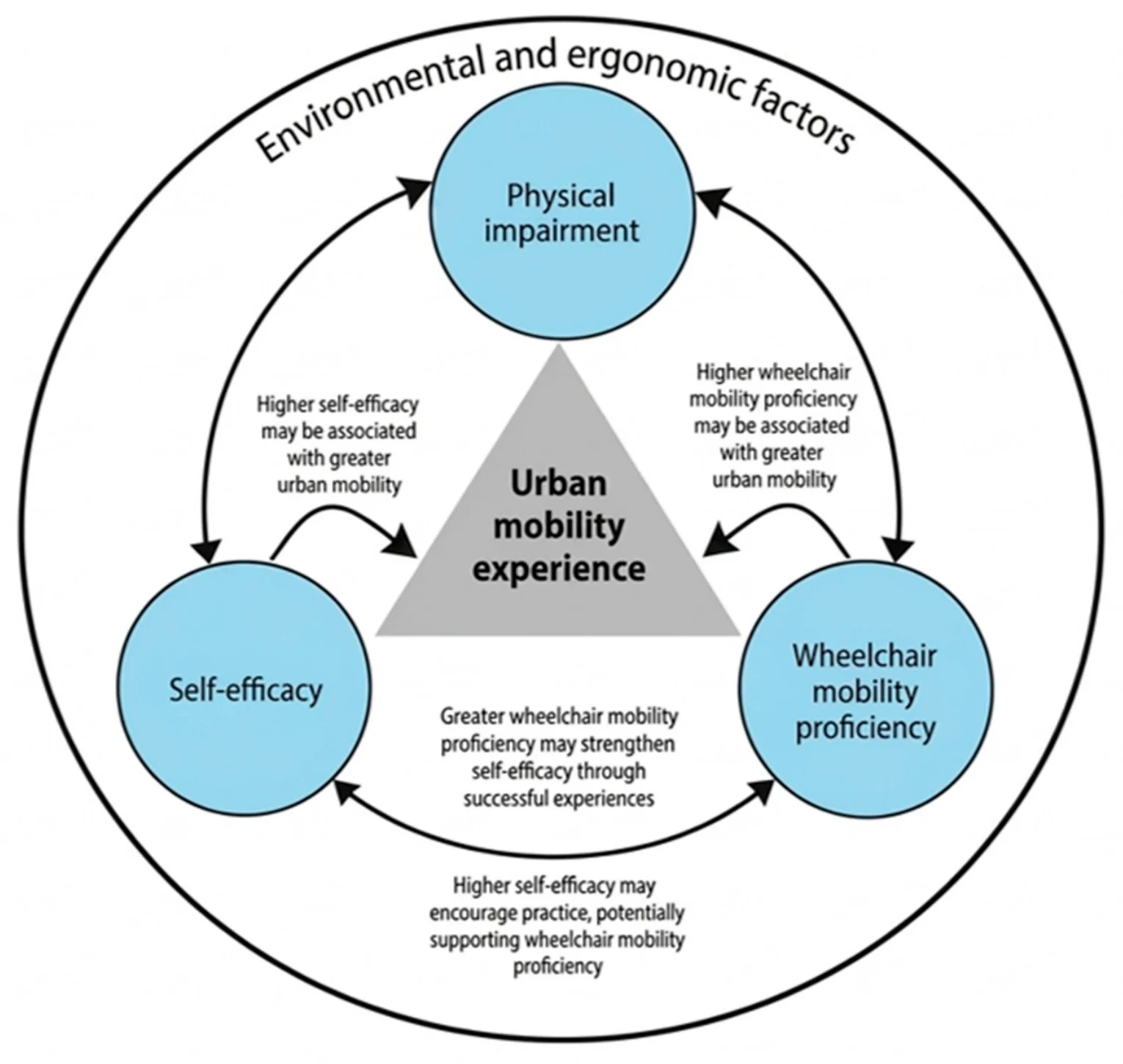
Proposed ICF-guided conceptual framework illustrating hypothesized relationships among PI, self-efficacy, and wheelchair mobility proficiency may interact to shape manual wheelchair users’ urban mobility experience, under the influence of environmental and ergonomic factors. The arrows represent conceptual relationships inferred from findings across separate studies rather than relationships demonstrated simultaneously within the included literature. Physical impairment sets the baseline of functional ability; environment and ergonomics determine how effectively that functional ability can be used; wheelchair mobility proficiency shapes what is achievable in real-world environments; and self-efficacy governs willingness to engage.

Based on the separate findings across the included studies and the supporting literature discussed above, we propose a conceptual interpretation in which PI, self-efficacy in wheeled mobility, and wheelchair mobility proficiency may interact to shape manual wheelchair users’ urban mobility experiences. Higher levels of PI may be associated with greater physical and ergonomic demands of mobility tasks, which could reduce self-efficacy and increase the level of proficiency required to navigate barriers. Wheelchair mobility proficiency may, in turn, influence self-efficacy, as users with greater proficiency may feel safer and more autonomous, whereas limited proficiency may contribute to insecurity and task avoidance. However, these relationships were not examined simultaneously within any included study and should therefore be regarded as conceptual propositions requiring empirical investigation. Figure 3 illustrates this proposed conceptual synthesis and highlights how the three user-level dimensions may interact with environmental and assistive-device conditions.

This review has several limitations. Restricting eligibility to English-language publications may have excluded relevant evidence published in other languages, while the January 2015 to May 2025 publication window may have omitted earlier foundational studies. The review was limited to peer-reviewed journal articles and did not include gray literature, such as reports, theses, policy documents, or other non-indexed sources; as a result, relevant evidence may not have been identified. The heterogeneity of study designs, participant characteristics, and outcome measures also limited direct comparison across studies. Because participant-based and modeling studies provide different types of evidence, their findings should not be interpreted as equivalent. In addition, some review dimensions, particularly wheelchair mobility proficiency and self-efficacy in wheeled mobility, had to be inferred from broader constructs because standardized measures were not consistently used. Participation was not assessed as a distinct standardized outcome across the included studies; therefore, references to participation should be interpreted as possible implications of perceived barriers, self-efficacy, and wheelchair mobility performance rather than as direct evidence of participation. Critical appraisal findings were considered narratively when interpreting the evidence and identifying methodological gaps, but were not used to exclude or quantitatively weight the included studies. Furthermore, although wheelchair ergonomics and configuration are likely to influence physical demands, mobility proficiency, and self-efficacy, these factors were not defined as a separate review dimension and were therefore not examined comprehensively in the results. Because studies involving only powered-wheelchair users were outside the predefined scope, this review cannot determine which urban mobility barriers are shared across wheelchair types and which are specific to manual wheelchair use. Future comparative research, including both manual and powered wheelchair users, could address this gap. Finally, transforming qualitative findings into study-level categorical indicators required reviewer interpretation, although a structured coding framework and reviewer discussion were used to support consistency.

## 5. Conclusions

This scoping review synthesized evidence on how urban accessibility challenges vary for manual wheelchair users by PI, self-efficacy in wheeled mobility, and wheelchair mobility proficiency. While each factor has been studied separately, the literature remains fragmented, with no study examining all three within a single framework, limiting the understanding of how user dimensions and environmental conditions jointly relate to everyday mobility.

Overall, greater PI was associated with more perceived or reported barriers, higher effort, and greater vulnerability to environmental shortcomings. Self-efficacy was associated with whether users attempted or avoided mobility tasks and may be relevant to participation beyond physical functioning alone. Wheelchair mobility proficiency, supported by training, experience, and wheelchair fit, affected users’ ability to manage real-world demands such as ramps, uneven surfaces, and public transportation. Some included studies also suggested that wheelchair fit and configuration may influence mobility performance, although these factors were not examined comprehensively as a distinct review dimension. Together, these dimensions may interact in reinforcing cycles that either promote autonomy or deepen exclusion; however, these relationships require direct empirical investigation.

Aligned with the ICF, the findings support the view that accessibility should be understood through interactions between body functions and structures, activities and participation, personal factors, and environmental conditions. It cannot be understood solely through environmental assessment or reduced to clinical severity; it reflects the match (or mismatch) between PI, self-efficacy, wheelchair mobility proficiency, assistive device characteristics, and the built environment.

Key gaps remain: inconsistent definitions and measures limit comparability; objective, standardized wheelchair mobility proficiency assessments are scarce; macro-level determinants are rarely integrated; and research is concentrated within a small number of national groups, underscoring the need for broader international collaboration.

Future research could benefit from integrative designs that assess all three dimensions using standardized measures, while also accounting for wheelchair-related dimensions, including ergonomic fit and configuration. Such studies should combine self-reported and qualitative outcomes with objective measures of wheelchair mobility, such as standardized wheelchair-skills assessments, travel speed and distance, slope negotiation, propulsion characteristics, physical effort, and rolling resistance. For policy and practice, efforts to improve participation may need to combine infrastructure adaptation, wheelchair skills training, self-efficacy-building strategies, and equitable access to well-fitted assistive devices.

In conclusion, urban accessibility is not only a matter of ramps and regulations; it should be understood in relation to physical functioning, self-efficacy, wheelchair mobility proficiency, assistive technology, and environmental design.

## Figures and Tables

**Table 1:** ICF-informed coding framework used for data charting and synthesis.

Review Dimension	ICF Component	How It Was Operationalized in Included Studies
Physical impairment	Body functions and structures	Type and level of physical impairment, including functional limitations that may influence how urban mobility barriers are experienced
Wheelchair mobility proficiency	Activities and participation	Maneuvering ability and performance in wheelchair mobility tasks, shaped by training, experience, and exposure to mobility challenges
Self-efficacy in wheeled mobility	Personal factors	Perceived ability or confidence to perform wheelchair-related mobility tasks or to face environmental mobility challenges
Urban environment and wheelchair/device factors	Environmental factors	Built-environment, transport, climate, and wheelchair/device characteristics that may act as barriers or facilitators to mobility

**Table 2:** Distribution table of articles based on the criteria.

Study	Study Design	Physical Impairment	Self-Efficacy in Wheeled Mobility	Wheelchair Mobility Proficiency
[25]	Observational study	x		x
[28]	Applied study and methodological model		x	
[35]	Qualitative study	x		x
[36]	Cross-sectional online survey study	x	x	
[37]	Cross-sectional	x		
[38]	Explorative qualitative study	x		
[26]	Obtain wheelchair users’ reports			x
[16]	Cross-sectional, web-based survey	x		
[39]	Single-arm pre–post feasibility pilot		x	
[40]	Mixed-methods—semi-structured interviews + video-observations	x		
[41]	Cross-sectional online survey	x		
[29]	Using a simulated manual wheelchair user in a real urban pedestrian network. No empirical participant dataset is collected; user parameters are simulated.		x	
[27]	Geographic Information Systems (GIS)-based modeling and simulation case study		x	
[42]	Methodology development and proof-of-concept case study		x	

**Table 3:** Characterization of the articles.

Physical Impairment						
Study	Sample	Environment or Context	Main Challenges	Facilitators	Concept or Measurement *	Key Findings
[25]	414 wheeled mobility device users (68 manual wheelchair, 312 power wheelchairs, 34 scooter).	Public transport (bus ramps).	Boarding and alighting incidents on ramps.	NR	Based on device type.	Manual wheelchair users had greater difficulty compared to scooter and power wheelchair users.
[35]	48 wheelchair users.	Public transport, urban routes, public buildings.	Uneven sidewalks; lack of ramps; poor wheelchair fit; instability; stigma.	Social support; training; accessible services.	Functional variation described qualitatively.	Users with more complex mobility needs reported greater instability, pain, and difficulty maneuvering unsuitable wheelchairs.
[36]	1219 university students with impairment.	Physical activity or university environment.	Environmental barriers (potholes, accessibility issues).	Family and friends support; professional guidance; adapted programs; perceived benefits.	Impairment grade (%) in official certificate.	Higher impairment grade: more perceived barriers; intrapersonal most frequent.
[37]	2041 adults with impairment.	Community or urban environment.	Difficulty commuting to health centers, schools, workplaces, social activities.	NR	Self-reported: mild, moderate, severe, extreme.	Higher severity strongly associated with perceived barriers (Moderate OR 2.39; Severe and Extreme OR 4.58).
[38]	16 adults with SCI (paraplegia = 11; tetraplegia = 5).	Public buildings, sidewalks, transport, rural and urban mobility.	Inaccessible restrooms; steep ramps; heavy doors; lack of curb cuts; inaccessible buses and trains.	Some accessible malls; peer support; adapted private vehicles.	Based on SCI level (para vs. tetra).	Participants with tetraplegia reported greater dependence, more difficulty with transfers, hygiene, and daily activities.
[16]	181 manual wheelchair users.	Physical activity or community.	Barriers in transport, sidewalks, fitness centers.	NR	BPAQ-MI questionnaire (0–5 scale).	SCI presence or level not associated with barrier domains.
[40]	69 adults with mobility impairments.	Indoor home settings; local travel; public transport.	Transfers; inaccessible bathrooms; narrow spaces; unstable devices; climate-related mobility issues.	NR	Severity based on extent of impairment (limbs and body parts affected).	More extensive impairment (legs + hands; full paralysis) reported greater difficulty with transfers and upper-limb control.
[41]	105 individuals with mobility impairments.	Urban pathways (sidewalks, slopes, crossings).	Lack of sidewalks; steep slopes; long distances; inadequate crossings.	NR	Severity inferred from mobility device type (prosthesis, MWC, PWC).	Manual wheelchair users reported highest dissatisfaction; environmental barriers more impactful for MWC users.
**Self-Efficacy in Wheeled Mobility**						
**Study**	**Sample**	**Environment or** **Context**	**Main Challenges**	**Facilitators**	Concept or Measurement *	**Key Findings**
[28]	127 manual wheelchair users	Pedestrian network (sidewalks, crosswalks, footpaths)	Uneven and cracked surfaces; potholes; steep slopes; snow; gravel and grass; curb up and down; crowds; crossings with time pressure	Wider sidewalks; adequate ramps; unobstructed paths	Perceived capability; WheelCon (0–100)	Uneven and obstructed segments showed low self-efficacy even when passable
[36]	1219 Spanish university students with impairment	Community & outdoor PA environment	Lack of self-efficacy for PA; inaccessible sidewalks; potholes; non-adapted crossings and lights; dangerous traffic; lack of adapted transport	Social support; adapted programs and facilities	Intrapersonal barrier; BPAQ-MI (single item)	self-efficacy was low and embedded among other PA barriers
[39]	n = 12 enrolled; n = 11 post (T2); n = 9 follow-up (T3)	Remote PA intervention (non-built environment audit)	Inaccessible facilities; transport barriers; health complexity; cost	Peer coaching; telehealth; goal planning	Self-efficacy; LTPA Barrier SE Scale (0–6)	No significant change T1→T2 (4.2→4.5, *p* = 0.13); decrease T2→T3 (*p* = 0.01)
[29]	Demonstration for 2 wheelchair users (self-efficacy values simulated, not measured)	Urban pedestrian network (sidewalk segments)	Steep slopes; narrow width; poor surface quality	Gentle slopes; good surface; sufficient width	Perceived ability; fuzzy sets	Higher self-efficacy increased modeled accessibility
[27]	self-efficacy data from >120 MWC users; map described as based on 127 users	Quebec City pedestrian network	Snow; trash bins; steep or narrow curb cuts; narrow sidewalks; poor surfaces; poles	Snow removal; curb-cut improvements	WheelCon (fuzzy categories)	Higher self-efficacy expanded accessible network
[42]	3 manual wheelchair users	Urban pedestrian network (routing analysis)	High curbs and steps; uneven surfaces; long inclines; snow; crowds	Gentle slopes; wider sidewalks; good surface	WheelCon	Routes differed according to self-efficacy profiles
**Wheelchair Mobility Proficiency**						
**Study**	**Sample**	**Environment or Context**	**Main Challenges**	**Facilitators**	Concept or Measurement *	**Key Findings**
[25]	414 wheeled mobility device users (68 MWC, 312 PWC, 34 scooters)	Public transport (bus ramps)	Maneuvering ramps at steep slopes; aligning device with ramp; repeated attempts; tipping risk	Fully kneeling the bus; deploying ramp to sidewalk or curb; operator awareness; automated alerts for excessive slope	Skill conceptualized as ability to maneuver WhMD on ramps and during boarding or getting off; no formal measurement	Ramp slopes exceeding 9.5° were associated with more incidents and assistance, while approach or departure angles greater than 30° were involved in several incidents. Wheelchair mobility proficiency was not formally measured.
[35]	48 adult wheelchair users	Urban routes, public transport, public buildings	Navigating uneven sidewalks; solving mobility problems; using public bathrooms; managing obstacles	Social support; peer networks; accessible services; attitudinal change	Skills conceptualized as maneuvering, problem-solving, and handling environmental barriers; no formal measurement	Lack of training reduced independence and safety; users with more training reported better functioning; wheelchair mobility proficiency shaped by experience and exposure
[26]	384 wheelchair users	Public transit systems (bus ramps)	Managing steep slopes, wet surfaces, thresholds; ingress or egress difficulties	Kneeling the bus; deploying ramp to elevated surfaces; operator training; improved ramp design	No formal skill test; skill inferred from difficulty or incident reports	Users without training reported greater difficulty; environmental demands (slope, wetness) often exceeded users’ skill capacity; even trained users struggled under challenging conditions

* We use the terms found in the original texts. NR = Not Reported; SCI = Spinal Cord Injury; BPAQ-MI = Barriers to Physical Activity Questionnaire for People with Mobility Impairments; MWC = Manual wheelchair; PWC = Power wheelchair; OR = Odds Ratio; WheelCon = Wheelchair Use Confidence Scale; PA = physical activity; LTPA Barrier SE Scale = Leisure-Time Physical Activity Barrier Self-Efficacy Scale; WhMD = Wheeled Mobility Device.

## Data Availability

No new data were created or analyzed in this study. Data sharing is not applicable to this article.

## Supplementary Materials

The following supporting information can be downloaded at: https://www.mdpi.com/article/10.3390/healthcare14162517/s1, Table S1: Search log; Table S2: Data Extraction; Table S3: Full Appraisal Results; Table S4. PRISMA-ScR checklist.

## Abbreviations

The following abbreviations are used in this manuscript:

BASE	Bielefeld Academic Search Engine
BPAQ-MI	Barriers to Physical Activity Questionnaire for People with Mobility Impairments
CI	Confidence interval
GIS	Geographic Information Systems
HH	Seyedeh Hiva Houshyar Yazdian
ICF	International Classification of Functioning, Disability and Health
JBI	Joanna Briggs Institute
KS	Karina Santos Guedes de Sá
LTPA	Leisure-time physical activity
MEDLINE	Medical Literature Analysis and Retrieval System Online
MeSH	Medical Subject Headings
MWC	Manual wheelchair
NGO	Non-Governmental Organization
NR	Not reported
OR	Odds ratio
OSF	Open Science Framework
PA	Physical activity
PCC	Population, Concept, Context
PI	Physical impairment
PRISMA-ScR	Preferred Reporting Items for Systematic Reviews and Meta-Analyses extension for Scoping Reviews
PWC	Power wheelchair
SCI	Spinal cord injury
SE	Self-efficacy
T1	Baseline assessment
T2	Post-intervention assessment
T3	Follow-up assessment
WheelCon	Wheelchair Use Confidence Scale
WhMD	Wheeled mobility device
YV	Yves Vanlandewijck
